# Epilepsy as primary tumor manifestation correlates with patient status, age, and tumor volume but not with survival in elderly glioblastoma patients: a retrospective bicentric analysis

**DOI:** 10.1007/s10143-025-03397-1

**Published:** 2025-02-24

**Authors:** Matthias Demetz, Constantin Hecker, Hamza Adel Salim, Aleksandrs Krigers, Jürgen Steinbacher, Lukas Machegger, Johannes Kerschbaumer, Melanie Buchta, Johannes Pöppe, Philipp Geiger, Antonio Spinello, Theo F. J. Kraus, Christoph J. Griessenauer, Claudius Thomé, Christian F. Freyschlag, Christoph Schwartz

**Affiliations:** 1 Department of Neurosurgery, Medical University of Innsbruck, Anichstr. 35, AT-6020, Innsbruck, Austria; 2 Department of Neurosurgery, University Hospital Salzburg, Paracelsus Medical University, Ignaz-Harrer-Strasse 79, AT-5020 Salzburg, Austria; 3 Department of Medicine, An-Najah National University, Nablus, Palestine; 4 Department of Radiology, Division of Neuroradiology, Johns Hopkins Medical Center, Baltimore, MD USA; 5 Department of Neuroradiology, University Hospital Salzburg, Paracelsus Medical University, Salzburg, Austria; 6 Institute of Pathology, University Hospital Salzburg, Paracelsus Medical University, Salzburg, Austria

**Keywords:** Elderly, Epilepsy, Glioblastoma, Outcome, Seizures

## Abstract

Assessment of the potential impact of epilepsy as primary tumor manifestation on survival in elderly glioblastoma multiforme (GBM) patients. Two academic neurosurgical centers retrospectively analyzed all GBM patients aged 65 years and older with de-novo tumors, who underwent surgery between 2006 and 2021. Epidemiological, histopathological, imaging and survival data were obtained from patients’ electronic charts, and screened for the presence of epilepsy preoperatively and during follow-up. Patient status was assessed by the Karnofsky Performance Scale (KPS), epilepsy was evaluated using the Engel classification. Patients were categorized in an epilepsy cohort (i.e. patients with epilepsy as primary GBM manifestation, and a reference cohort (i.e. patients with no initial epilepsy). This study analyzed 451 GBM patients (55% males); median age at tumor diagnosis was 73 years. The epilepsy cohort was younger (71.0 vs. 74.0 year; p = 0.001), had better KPS (80 vs. 70; p = 0.039), and had smaller tumors (127 vs. 221 cm^3^; p = 0.001) compared to the reference cohort. There were no differences with regard to neurosurgical treatment (i.e. resection vs. biopsy), tumor biomarkers, surgery-associated complications, and performed adjuvant treatment (all p > 0.05). We did not detect a significant difference in median survival between the epilepsy and reference cohorts (8 vs. 6 months; p = 0.21). New epilepsy during follow-up often coincided with tumor recurrence/progression, but also did not significantly affect survival. The majority of patients (98%) achieved seizure freedom by a combination of tumor-specific treatments and antiseizure medication. Epilepsy as primary tumor manifestation does not correlate with survival in elderly GBM patients.

**Supplementary Information:**

The online version contains supplementary material available at 10.1007/s10143-025-03397-1.

## Introduction

Glioblastoma multiforme (GBM) WHO (World Health Organization) grade 4, the most common and malignant primary brain tumor, still poses a tremendous challenge to neuro-oncologists due to its intrinsic resistance to conventional therapies and its propensity for rapid progression.

Epilepsy is a common initial symptom in glioma patients. It was reported that epileptogenic gliomas might convey a more favorable prognosis because of earlier diagnosis and lower tumor volumes, especially in low-grade gliomas (LGG)[12, 31]. Furthermore, surgical resection has been shown to not only positively influence survival in LGG patients in previous studies, but also led to a higher incidence of postoperative seizure freedom[39]. More recently, evidence has emerged that epileptic activity is also associated with improved survival in patients suffering from high-grade gliomas (HGG) [25, 26, 28]. However, data on preoperative epileptic seizures and their impact on outcome, especially survival, in elderly GBM patients are limited. To the best of our knowledge, there are also no reports to this date on the relationship between seizure freedom and extent of resection (EOR) in elderly GBM patients.

The current study had two main goals. The primary objective was the analysis of the impact of epilepsy as primary tumor manifestation on survival in GBM patients aged 65 years and older. The secondary objective was the evaluation of potential correlation of new seizures over the course of the disease with patient outcome. Additionally, we assessed the incidence of seizure freedom and investigated the impact of surgical resection on postoperative seizures.

## Material & Methods

### Patient selection

In this retrospective study, we included all consecutive GBM patients aged 65 years and older at initial tumor diagnosis, who underwent their first surgery at two university hospitals between 2006 and 2021, for further analysis. All patients had given prior written informed consent to all applied treatments; no study-specific treatments were conducted.

Histopathological grading of the tumors was conducted based on the WHO classification of central nervous system tumors applicable at the time of diagnosis [17–19].

Clinical data were extracted from electronic medical records, which included patient characteristics such as age, sex, Karnofsky Performance Scale (KPS)[21], patient frailty assessed by the Modified 5-Item Frailty Index (mFI-5)[35], tumor location, and the main symptoms reported by the patients.

Treatment decisions were made by each center’s interdisciplinary tumor board according to the guidelines applicable at the time of diagnosis. Treatment was performed in accordance with the Declaration of Helsinki.

### Epilepsy assessment

The definition of preoperative epilepsy was based on the International League Against Epilepsy 2017 classification (ILAE-2017), which categorized seizures into focal onset aware or impaired awareness, generalized onset with bilateral tonic-clonic seizures as the main subtype, or status epilepticus (SE) [7, 29, 38]. Epilepsy outcomes were evaluated using the Engel classification [6]. The Engel classification includes four classes: Class I indicates complete seizure freedom since surgery, Class II represents rare or non-disabling seizures, such as nocturnal seizures, Class III denotes a significant reduction in seizures, and Class IV indicates no significant seizure reduction or even worsening. Seizure control was defined as Class IA at last follow-up and was used in our statistical analysis for better understanding. The presence of epilepsy and the course of epilepsy outcome were assessed clinically, routinely supported by electroencephalography, during outpatient clinic visits usually performed at 3-month intervals. Status epilepticus can have a significant impact on the quality of life (QoL) in glioma patients, as it often leads to prolonged neurological deficits, increased hospitalizations, and a greater burden on caregivers[1, 34]. Given its potentially more severe consequences, we performed a separate analysis of SE in our cohort to better understand its distinct effects compared to other types of seizures.

For analysis of the primary study objective, the analysis of epilepsy as primary tumor manifestation, the study population was divided into two distinct cohorts: Patients who presented with epilepsy as initial symptom of a GBM (i.e. epilepsy cohort), and patients with no epilepsy prior to the initial tumor diagnosis (i.e. reference cohort). As for the secondary outcome parameter, the evaluation of the impact of newly developed epilepsy during follow-up, patients were categorized into those who suffered from newly diagnosed seizures over the course of the disease and did not suffer from epilepsy as a primary tumor manifestation (i.e. follow-up manifestation cohort), and those who never experienced any seizures. The follow-up cohort is, thus, a subgroup of the reference cohort. Further post hoc analyses included analyses of seizure type and survival.

### Imaging

For most cases, both, preoperative and early postoperative (within 72 hours after surgery) magnetic resonance imaging (MRI) data were available. Two members (JS and LM) of the Institute of Neuroradiology evaluated all available pre- and immediate postoperative MRIs, and tumor volumes as well as the extent of resection (EOR) were semiautomatically and three-dimensionally assessed based on contrast-enhanced MRI as previously described[30]. The EOR was calculated as the percentage of resected contrast-enhancing tumor volume.

### Statistics

Statistical analyses were performed using R Studio Version 4.2.2. Categorical variables were summarized as frequencies and percentages, compared using the χ2 test and Fisher’s exact test where sample sizes were less than five. Continuous variables were presented as medians and Interquartile range (IQRs), compared using the Mann-Whitney U test. Survival outcomes were analyzed using the Kaplan-Meier method. The reference date for all survival analyses was the date of tumor resection/biopsy; overall survival was calculated using either the date of the patient’s death or the date of the last documented follow-up visit. Differences in survival distributions were evaluated using the log-rank test. To determine the impact of seizure/SE manifestation on survival and functional status, unadjusted and adjusted Cox proportional hazards models were used. Ordinal logistic regression was applied to assess the KPS at different time points. The models were adjusted for potential confounders, including age, tumor location, depth, eloquence, frailty index, tumor volume, and preoperative KPS. The hazard ratios (HRs) and odds ratios (ORs), along with their 95% confidence intervals (CIs), were reported. Further subgroup analyses were performed based on seizure type (focal vs. generalized vs. SE). Both unadjusted and adjusted models were utilized. All statistical tests were two-sided, and a p-value of less than 0.05 indicated statistical significance.

## Results

### Patient characteristics

A total of 451 patients were included; the epilepsy cohort consisted of 112 patients, and the reference cohort of 339 patients (Table 1). The median age of all patients was 73.0 years (IQR 69.0–77.0), with a significant age difference observed between cohorts (*p* = 0.001); patients with epilepsy as primary tumor manifestation were younger at diagnosis compared to those in the reference cohort (median 71.0 years (IQR 68.0–75.0) vs. median 74.0 years (IQR 70.0–78.0)). Gender distribution showed a slight majority of male patients (55.0%) as previously described for gliomas, and no significant difference in distribution between study cohorts (*p* = 0.52). Recorded comorbidities showed no significant differences across groups (data not shown) and no significant difference in the mFI-5 between study groups was recorded (p = 0.21). The median preoperative KPS was higher in the epilepsy cohort (80 [IQR; 70–90] vs. 70 [IQR; 70–90]; *p* = 0.039). The higher KPS was most likely due to a significantly lower percentage of focal neurological deficits, notably hemiparesis (14 (16.0%) additionally to seizures vs. 122 (43.0%)) in the control group, as a primary manifestation in the reference group (Table 1). In Table 1, the seizure characteristics also include the follow-up period, which explains why some patients in the reference cohort developed seizures over time. Among the epilepsy cohort, 48 (43.0%) patients presented with focal seizures, and 64 (57.0%) with generalized seizures. Additionally, twelve patients were admitted to the hospital with an initial SE; eleven suffered from a convulsive SE and one patient from a non-convulsive SE (NCSE).

**Table 1:** Patient and tumor characteristics

Characteristic	All patients, *N* = 451	Reference cohort, *N* = 339	Epilepsy cohort, *N* = 112	*p*-value^*1*^
Age, Median (IQR)	73.0 (69.0, 77.0)	74.0 (70.0, 78.0)	71.0 (68.0, 75.0)	0.001
Sex, *n* (%)				0.52
Male	246 (55)	182 (54)	64 (57)	
Female	205 (45)	157 (46)	48 (43)	
Preoperative KPS, Median (IQR)	80 (60, 90)	70 (60, 90)	80 (70, 90)	0.039
mFI-5, Median (IQR)	1 (0, 1)	1 (0, 1)	1 (0, 1)	0.21
Seizures, *n* (%)^2^				< 0.001
Focal	66 (15)	18 (5.3)	48 (43)	
Generalized	88 (20)	24 (7.1)	64 (57)	
None	297 (66)	297 (88)	0 (0)	
Convulsive Status epilepticus, *n* (%)^2^				0.034
None	421 (93)	321 (95)	100 (89)	
NCSE	7 (1.6)	6 (1.8)	1 (0.9)	
Convulsive SE	23 (5.1)	12 (3.5)	11 (9.8)	
Preoperative hemiparesis/hypesthesia, *n* (%)				< 0.001
Hemiparesis	136 (36)	122 (43)	14 (16)	
Hemihypesthesia	12 (3.2)	7 (2.4)	5 (5.7)	
None	227 (61)	158 (55)	69 (78)	
Preoperative tumor volume [cm3], Median (IQR)	201 (89, 314)	221 (111, 316)	127 (55, 298)	0.001
Most common locations, n (%)				
temporal	138 (31)	99 (29)	39 (36)	
frontal	118 (26)	88 (26)	30 (27)	
parietal	39 (8.6)	28 (8.2)	11 (11)	
multilocular	27 (5.9)	22 (6.4)	5 (5.4)	
Deep location, *n* (%)	129 (29)	102 (30)	27 (25)	0.33
Eloquent location, *n* (%)	236 (52)	172 (51)	64 (57)	0.24
Side, *n* (%)				0.31
right	229 (51)	177 (52)	52 (46)	
left	219 (49)	159 (47)	60 (54)	
bilateral	3 (0.6)	3 (0.9)	0 (0)	
Histology, *n* (%)				> 0.99
GBM WHO IV	446 (99)	335 (99)	111 (99)	
Gliosarcoma WHO IV	5 (1.1)	4 (1.2)	1 (0.9)	
IDH mutation status, *n* (%)				> 0.99
IDH1 Mutation	3 (0.9)	2 (0.8)	1 (1.2)	
IDH wildtype	316 (99)	234 (99)	82 (99)	
MGMT methylation status, *n* (%)				0.14
Methylated	135 (37)	96 (35)	39 (42)	
Unmethylated	104 (28)	75 (27)	29 (32)	
Partial methylation	126 (35)	102 (37)	24 (26)	

^*1*^ Wilcoxon rank sum test; Pearson’s Chi-squared test; Fisher’s exact test

^*2*^ The epilepsy characteristics also take into account the seizures/status epilepticus that occurred during the follow-up in the reference cohort.

The follow-up manifestation cohort, i.e. patients who developed new epilepsy during the course of the disease, consisted of 50 patients. Of those, 38 (76.0%) were treated by microsurgery and 12 (24.0%) patients underwent biopsy.

### Tumor characteristics

The most common overall tumor locations were the temporal lobe in 138 (31.0%), followed by the frontal lobe in 115 (26.0%), and the parietal lobe 39 (8.9%) patients; 27 (6.1%) tumors showed a multilocular growth pattern at initial diagnosis and 129 (29%) tumors were categorized as deep-seated (Table 1). No difference between groups could be detected with regard to deep-seated or eloquent location as well as tumor lateralization (*p* = 0.33, *p* = 0.24, and *p* = 0.31, respectively) (Table 1). The median contrast-enhancing tumor volume was 201 cm^3^ (IQR 89– 314); tumor volumes were significantly smaller in patients with primary manifestation of seizures (127 vs. 221 cm³; *p* = 0.001) (Table 1). Biomarker status analyses including isocitrate dehydrogenase (IDH) mutation (*p* > 0.99) and O6-methylguanine-DNA methyltransferase (MGMT) promoter methylation (*p* = 0.14) status showed no difference between the two cohorts (Table 1).

### Treatment and surgery-associated complications

Overall, 68.0% of patients underwent microsurgical tumor resection and 32.0% only had a biopsy as neurosurgical treatment; no significant difference with regard to neurosurgical treatment was seen between the epilepsy and reference cohorts (*p* = 0.77). Among patients treated by microsurgery, no significant difference in extent of resection was recorded (*p* = 0.54) (Table 2). Importantly, no difference in the frequency of performed adjuvant therapy between groups was noted (*p* = 0.25). Furthermore, there was no difference in recorded surgical complications (*p* = 0.54), while the four most common complications were new focal neurological deficit with 11.0%, followed by hemorrhage (2.6%), organic psychosyndrom (2.3%), and abscess (1.2%).

**Table 2:** Treatment and outcome

Characteristic	All patients, *N* = 451	Reference cohort, *N* = 339	Epilepsy cohort, *N* = 112	*p*-value^*1*^
*Treatment details*				
Type of surgery, *n* (%)				0.77
Only biopsy	146 (32)	111 (33)	35 (31)	
Resection	305 (68)	228 (67)	77 (69)	
Extent of resection, Median (IQR)	95 (86, 98)	95 (86, 98)	94 (85, 98)	0.42
Surgical complications, *n* (%)	111 (25)	81 (24)	30 (27)	0.54
Most common surgical complications type, *n* (%)				0.15
New deficit	46 (11)	33 (10)	13 (12)	
Hemorrhage	11 (2.6)	10 (3.1)	1 (0.9)	
Organic psychosyndrome	10 (2.3)	6 (1.9)	4 (3.8)	
Wound healing deficiency	6 (1.4)	5 (1.6)	1 (0.9)	
Outcome				
Epilepsy post-surgery, *n* (%)				< 0.001
Improved	65 (37)	1 (1.3)	64 (64)	
Worsened	14 (8.0)	9 (12)	5 (5.0)	
Unchanged	96 (55)	65 (87)	31 (31)	
Controlled Seizure, *n* (%)	92 (93)	12 (71)	80 (98)	0.001
Postoperative outcome hemiparesis/hypoesthesia, *n* (%)				0.64
Improved	51 (26)	43 (27)	8 (24)	
Worsened	36 (18)	28 (17)	8 (24)	
Unchanged	108 (55)	91 (56)	17 (52)	
New deficit after surgery, *n* (%)	62 (14)	47 (14)	15 (14)	0.92
Postoperative KPS, Median (IQR)	80 (70, 90)	80 (70, 90)	90 (80, 90)	0.001
KPS last follow-up before death, Median (IQR)	60 (50, 80)	60 (50, 80)	70 (60, 80)	0.13

^*1*^ Wilcoxon rank sum test; Pearson’s Chi-squared test; Fisher’s exact test

### Functional outcome, survival, and impact of epilepsy

The median postoperative KPS was 80 (IQR 70–90); however, as the disease progressed, the patients’ functional status worsened accordingly with a median KPS of 60 (IQR 50–80) at the last follow-up before death. Postoperative functional status was better in the epilepsy cohort (90 (IQR 80–90) vs. 80 (IQR 70–90); *p* = 0.001) (Table 2). Postoperatively, 51 (26.0%) patients showed an improvement of hemiparesis/hypoesthesia symptoms with no difference between study groups (*p* = 0.64) (Table 2). Among the epilepsy cohort cases, seizure control was achieved in 98% of affected patients with or without antiepileptic medication. Monotherapy was adequate in 79.5% of cases, whereas 20.5% of cases required the use of more than one ASM, including two instances where triple medication was necessary. Levetiracetam was the most frequently used ASM (81.3%), followed by Carbamazepine (12.5%). For add-on therapy, Clobazam (7.1%) and Lacosamide (5.4%) were the most commonly used drugs. Among patients (*n* = 77), who underwent tumor resection and suffered from preoperative epilepsy, in 67 (99.0% of all documented cases) patients seizure-freedom was achieved; the corresponding of rate of seizure freedom in the biopsy group was 93.0% (14 documented cases out of 35 cases; *p* = 0.31). Post hoc analyses did not show a correlation of EOR and postoperative seizure freedom in resected cases (*p* = 0.328).

**Fig. 1:**
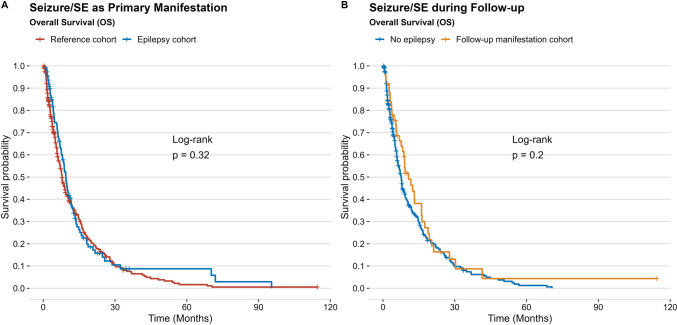
**A** – **B**: Kaplan-Meier analysis revealed no significant differences regarding OS between the two study cohorts

**Fig. 2:**
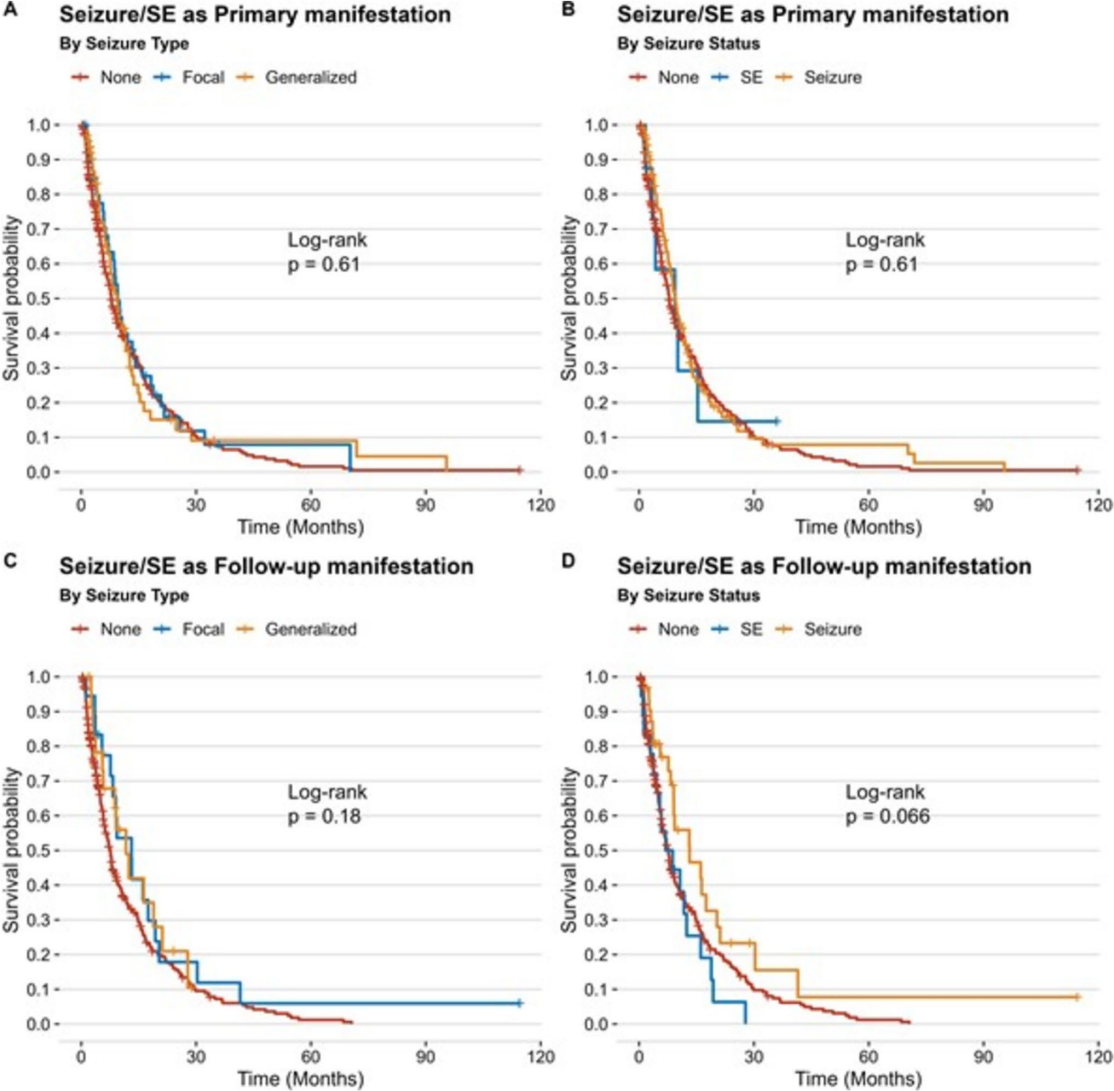
**A** – **D**: No significant differences for seizure type could be shown in Kaplan-Meier analysis

Within a median follow-up after neurosurgery of 6 months (IQR 3–13), 401 (88.9%) patients were confirmed to have died with a recorded date of death. In 50 (11.1%) cases, the date of death were missing and date of last follow up was used for survival analysis; there was no significant difference with regard to the frequency of unknown dates of death between the epilepsy and reference cohorts (p = 0.569). We found no significant survival differences between the epilepsy and reference cohort with recorded median OS of 6 (IQR 3–12) and 8 (IQR 3–13) months, respectively (*p* = 0.21; log-rank) (Fig. 1). Additionally, no significant differences in patient survival stratified by seizure type as primary or follow-up manifestation were recorded (Fig. 2). Regression analysis, adjusted for potential confounders such as age, deep-seated tumor location and eloquence, adjuvant therapy, frailty, tumor volume, and preoperative KPS, also confirmed that epilepsy as primary tumor manifestation did not significantly impact OS in our patient population (OR 0.87 (95% CI 0.68–1.13); *p* = 0.31) (Table 3).

**Table 3:** Outcomes cox regression and ordinal logistic regression model

Outcome	CSE/Seizures Manifestation Time	Unadjusted Model		Adjusted Model*	
		HR/OR (95% CI)^1^	*p*-value	HR/OR (95% CI)^1^	*p*-value
OS	None	*Reference*		*Reference*	
	Primary Manifestation	0.85 (0.66 to 1.09)	0.19	0.87 (0.68 to 1.13)	0.31
	Follow-up Manifestation	0.78 (0.55 to 1.10)	0.15	0.78 (0.55 to 1.11)	0.17
Preoperative KPS	None	*Reference*		*Reference*	
	Primary Manifestation	1.55 (1.06 to 2.28)	0.024	1.45 (0.98 to 2.13)	0.062
	Follow-up Manifestation	1.39 (0.83 to 2.35)	0.22	1.33 (0.79 to 2.26)	0.29
Postoperative KPS	None	*Reference*		*Reference*	
	Primary Manifestation	1.98 (1.31 to 3.00)	0.001	1.80 (1.19 to 2.75)	0.006
	Follow-up Manifestation	1.12 (0.65 to 1.93)	0.68	1.05 (0.61 to 1.82)	0.86
Last FU KPS	None	*Reference*		*Reference*	
	Primary Manifestation	1.27 (0.86 to 1.87)	0.22	1.30 (0.88 to 1.91)	0.19
	Follow-up Manifestation	0.69 (0.39 to 1.22)	0.2	0.70 (0.39 to 1.24)	0.22

^*1*^ *HR *Hazard Ratio,* OR *Odds Ratio,* CI *Confidence Interval

* All estimates were adjusted for patient age, tumor location depth and eloquence, adjuvant therapy, frailty index, tumor volume, and preoperative KPS (was not included in " Preoperative KPS")

As for the significance of newly developed seizures during follow-up, regression analyses did not detect a difference in OS between the follow-up manifestation cohort and patients who never experienced an epileptic seizures (OR 0.78 (95% CI 0.55–1.11); *p* = 0.17) (Table 3). However, seizure manifestation during follow-up correlated with tumor progression within 1 month of diagnosed tumor progress on MRI in 37.2% of cases. Post hoc analyses of seizure types including SE showed no correlations with OS and patients’ pre- and postoperative functional status (Table 3). Details of the follow-up manifestation cohort are shown in Supplementary Tables 1–3.

## Discussion

### Key findings

Our study’s main findings were, 1) elderly GBM patients with epilepsy as the initial tumor manifestation were younger, had better preoperative functional status, and smaller tumors compared to the reference cohort; 2) however, this did not translate into improved OS. Furthermore, 3) despite the fact that in patients, who experienced new epilepsy over the course of the disease, this often coincided with tumor recurrence/progression, these patients did not show worse outcome than patients who never developed epilepsy.

### Impact of epilepsy on outcome

To the best of our knowledge, this study is one of the largest focusing on the impact of epilepsy on outcome in elderly GBM patients to date. As life expectancy in developed countries continues to rise, the incidence of glioma among elderly patients is expected to increase significantly in the coming years [15]. This demographic shift may lead to a doubling of GBM cases in patients aged 65 and older over the next two decades, with elderly patients projected to account for two-thirds of all GBM cases by 2030, as estimated by the US National Institute on Aging [2, 8]. Therefore, acquisition of a better understanding of additional prognostic factors, in elderly patients with GBM is crucial.

While GBM can affect individuals of all age groups, it is particularly menacing when it afflicts elderly patients, usually defined as those aged 65 years and older [22]. Elderly GBM patients often encounter a multitude of factors that complicate their clinical management leading to an especially poor prognosis [9, 13, 14, 20]. This might not only be due to less resilience against aggressive multimodal tumor treatment, overall lower rates of performed adjuvant therapy, and increased risk for treatment-associated complications, but also due to more unfavorable molecular tumor characteristics. However, a possible undertreatment in these often-frail patients has already been postulated in literature [40]. Furthermore, managing epilepsy in neuro-oncological patients presents unique difficulties, since treatment with anti-seizure medication (ASM) alone might not be sufficient. Seizures can be both a presenting symptom and a complication of treatment, significantly impacting patients’ QoL and complicating therapeutic strategies [5, 32]. Balancing effective seizure control with the management of GBM poses a complex clinical conundrum, highlighting the imperative for tailored, multidisciplinary approaches to address the needs of this vulnerable patient population.

In our study, we uncovered findings that deviate from established patterns seen in LGG or younger patient cohorts. Contrary to expectations, we observed that preoperative epilepsy did not significantly affect OS in this demographic. This was somewhat surprising because in our study population, patients in the epilepsy cohort had better functional status, were younger, and had smaller tumors, all factors usually associated with improved survival in HGG and GBM patients. Also, our finding regarding the primary study objective is contrary to recently published results [24–26, 28]. Recent publications primarily included patients of younger age and therefore could not be directly comparable to our cohort; however, these studies also reported a younger age in epileptic GBM patients, aligning with our findings[26]. Importantly, in our analyzed patient population, there was no significant difference with regard to performed neurosurgical procedure, i.e. resection vs. biopsy, achieved EOR by tumor resection, surgery-associated complications, and performance of adjuvant treatment (Table 2). It has to be acknowledged that in the analyzed patient population survival was generally poor with a median OS of 6 months. However, taking into account that this study focused specifically on elderly patients with a median age at initial diagnosis of 73 years, the recorded OS is in line with previous reports on survival in old GBM patients. Elderly GBM patients commonly face a limited prognosis characterized by limited response to treatment modalities with a limited overall survival of less than a year in most cases [3, 16, 36]. Thus, the most likely explanation for our results is that our patients were significantly older than in other reports on epilepsy and outcome in HGG patients [24, 25]. By adjusting our survival analyses for known prognostic factors including patient age at initial diagnosis, we attempted to exclude potential confounders with regard to the prognostic impact of epilepsy as best as possible. Hence, it can be postulated that the aggressive tumor biology in combination with the advanced patient age outweighed the potential survival benefits of the epilepsy cohort in our patient population.

Nevertheless, the observation that GBM patients with preoperative seizures exhibited a significantly younger age at the time of diagnosis, along with significantly lower preoperative tumor volumes, suggests a diagnosis occurring at an earlier stage of the disease trajectory, what was already proposed in previous studies on LGG [11, 27]. This finding underscores the potential utility of epilepsy as an early clinical indicator in younger GBM cases, allowing for timely intervention and management strategies. The smaller tumor volumes observed in epileptic glioma patients may also facilitate surgical resection, as previously postulated in related studies, thereby potentially enhancing patient outcomes [23, 27], however, we were not able to support these claims in this elderly population. Another important aspect, which we were able to confirm in our analysis, was that a newly developed epilepsy over the course of the disease coincided with tumor recurrence/progression in a significant proportion (37.2%) of affected patients. Even though these patients did not show worse OS than patients who never suffered from epileptic seizures, new seizures should still always be seen as a warning sign and trigger cerebral imaging. Furthermore, a longer follow-up period may result in the observation of more seizures, as patients with extended survival are more likely to experience additional seizure events over time.

Amidst this challenging landscape, a great majority of patients (98%) experienced seizure control following tumor treatment as evidenced by an Engel 1 classification scoring. This finding not only demonstrate the potential therapeutic benefits of tumor-specific treatment in combination with ASMs in managing epilepsy in elderly GBM patients. This has already been described in previous studies on LGG [4, 11, 39], but also underscores the importance of considering QoL outcomes beyond traditional survival metrics. Moreover, our observation of significantly higher KPS among patients with preoperative seizures, both at the initial assessment and the last follow-up, suggests a tangible enhancement in functional status and overall well-being in this subgroup. This highlights the profound impact that successful epilepsy management can have on patients’ day-to-day functioning and underscores the holistic benefits of surgical intervention beyond merely extending survival. It further emphasizes the imperative of incorporating measures of functional status and QoL into the comprehensive evaluation and management of elderly GBM patients, aiming not only to prolong life but also to optimize its quality [10, 33, 37].

### Limitations

Our study has several limitations, mainly related to retrospective data collection and analysis. Even though treatment decisions were agreed upon in the participating centers’ tumor boards, there was no shared decision-making. Due to the retrospective design of the study patients who were diagnosed prior to the 2016 WHO classification were included, which may be considered a limitation as the tumor classification has since been updated. However, considering the small number of patients with IDH mutations in our cohort, we included these cases under the glioblastoma category to maintain consistency within our retrospective analysis. Epilepsy data were retrospectively extracted from electronic patient records, and therefore epilepsy assessment may have been incomplete in some cases leading to an under- or overestimation of the potential impact of epilepsy on the analyzed outcome parameters. Classifying seizures according to the updated ILAE classification is challenging in this retrospective cohort, as the data were collected prior to the introduction of the latest classification system. Furthermore, since all patients with seizures were treated with ASMs, which are usually not tapered off in GBM patients, the impact of tumor resection on postoperative seizure cannot be established with absolute certainty. No standardized QoL assessment was performed; thus, the positive impact of seizure freedom on patients’ QoL can only be assumed and our study falls short of providing a nuanced understanding of the subjective experiences and overall well-being of these individuals. Moving forward, prospective studies designed specifically to evaluate QoL in elderly GBM patients are warranted. By employing validated QoL assessment tools and incorporating patient-reported outcomes, such studies can offer more robust insights into the impact of preoperative seizures and other clinical factors on patients’ QoL, thereby informing more holistic and patient-centered approaches to care.

## Conclusion

Elderly GBM patients, who became symptomatic with an epileptic seizure, were of younger age, had better functional status, and suffered from smaller tumors compared to those with no initial seizure. Nonetheless, our analyses failed to confirm epilepsy as the initial tumor manifestation to be a prognostic factor for survival. Importantly, new seizures over the course of the disease often indicate tumor recurrence/progression in patients, who previously did not suffer from epilepsy. Furthermore, the majority of epilepsy patients achieved seizure freedom by a combination of tumor-specific treatments, including neurosurgery, and ASM.

## Supplementary Information

Below is the link to the electronic supplementary material.

ESM 1 (DOCX 33.6 KB)

ESM 2 (DOCX 23.6 KB)

## Data Availability

No datasets were generated or analysed during the current study.
