# Supplementary material for: Epilepsy as primary tumor manifestation correlates with patient status, age, and tumor volume but not with survival in elderly glioblastoma patients: a retrospective bicentric analysis
Source: Neurosurg Rev. 2025 Feb 24;48(1):264. doi: 10.1007/s10143-025-03397-1 (PMC11850559; doi:10.1007/s10143-025-03397-1)
Supplement: Supplementary file 2 — (DOCX 23.6 KB) [file 10143_2025_3397_MOESM2_ESM.docx]

Supplementary table 1: Patient and Tumor Characteristics

| **Characteristic** | **Follow-up manifestation cohort**  N = 50 |
| --- | --- |
| Age, Median (IQR) | 72.5 (70.0, 76.8) |
| Sex, n (%) |  |
| Male | 27 (54) |
| Female | 23 (46) |
| Preoperative KPS, Median (IQR) | 75 (70, 90) |
| mFI-5, Median (IQR) | 1 (0,1) |
| Seizures, n (%) |  |
| Focal | 18 (36) |
| Generalized | 24 (48) |
| None | 8 (16) |
| Status epilepticus, n (%) |  |
| None | 32 (64) |
| NCSE | 6 (12) |
| CSE | 12 (24) |
| Preoperative hemiparesis/hypesthesia, n (%) |  |
| Hemiparesis | 16 (37) |
| Hemihypesthesia | 2 (4.7) |
| None | 25 (58) |
| Volume [cm3] (1), Median (IQR) | 215 (123, 334) |
| Most common locations, n (%) |  |
| temporal | 10 (20) |
| frontal | 14 (28) |
| parietal | 6 (12) |
| multilocular | 3 (6.0) |
| Deep location, n (%) | 17 (34) |
| Eloquent location, n (%) | 26 (52) |
| Side, n (%) |  |
| right | 25 (50) |
| left | 23 (46) |
| bilateral | 2 (4) |
| Histology, n (%) |  |
| GBM WHO IV | 49 (98) |
| Gliosarcoma WHO IV | 1 (2.0) |
| IDH mutation status, n (%) |  |
| IDH1 Mutation | 1 (2.6) |
| IDH wildtype | 37 (97) |
| MGMT methylation status, n (%) |  |
| Methylated | 16 (37) |
| Unmethylated | 16 (37) |
| Partial methylation | 11 (26) |

Supplementary table 2: Treatment and outcome

| **Characteristic** | **Follow-up manifestation cohort**  N = 50 |
| --- | --- |
| ***Treatment details*** | |
| Type of surgery, n (%) |  |
| Only biopsy | 12 (24) |
| Resection | 38 (76) |
| Adjuvant therapy, n (%) | 44 (88) |
| Extent of resection, Median (IQR) | 95 (92, 97) |
| Surgical complications, n (%) | 16 (32) |
| Most common surgical complications type, n (%) |  |
| New deficit | 5 (11) |
| Hemorrhage | 3 (6.8) |
| Ischemia | 1 (2.3) |
| Wound healing deficiency | 1 (2.3) |
|  |  |
| **Characteristic** | **Follow-up manifestation cohort**  N = 50 |
| ***Outcome*** | |
| Epilepsy post-surgery, n (%) |  |
| Improved | 0 (0) |
| Worsened | 8 (36) |
| Unchanged | 14 (64) |
| Recurrent seizures, n (%) | 3 (23) |
| Controlled Seizure, n (%) | 12 (92) |
| Postoperative outcome hemiparesis/hypoesthesia, n (%) |  |
| Improved | 6 (21) |
| Worsened | 5 (18) |
| Unchanged | 17 (61) |
| New deficit after surgery, n (%) | 10 (21) |
| Postoperative KPS, Median (IQR) | 80 (75, 90) |
| KPS last follow-up before death, Median (IQR) | 60 (40, 80) |

Supplementary table 3: Subgroup analysis by seizures type

| **Outcome** | Seizures Status | SE/Seizures as Primary Manifestation | | | | SE/Seizures as Follow-up Manifestation | | | |
| --- | --- | --- | --- | --- | --- | --- | --- | --- | --- |
|  |  | **Unadjusted Model** | | **Adjusted Model*** | | **Unadjusted Model** | | **Adjusted Model*** | |
|  |  | **HR/OR** **(95% CI)***^1^* | **p-value** | **HR** **(95% CI)***^1^* | **p-value** | **HR/OR** **(95% CI)***^1^* | **p-value** | **HR** **(95% CI)***^1^* | **p-value** |
| OS | Focal | *Reference* | | *Reference* | | *Reference* | | *Reference* | |
|  | Generalized | 1.02 (0.67 to 1.57) | 0.91 | 1.04 (0.68 to 1.61) | 0.84 | 1.08 (0.53 to 2.19) | 0.84 | 1.08 (0.53 to 2.20) | 0.83 |
|  | None | 1.15 (0.82 to 1.61) | 0.43 | 1.12 (0.80 to 1.58) | 0.51 | 1.47 (0.88 to 2.45) | 0.14 | 1.46 (0.88 to 2.44) | 0.15 |
| Preoperative KPS | Focal | *Reference* | | *Reference* | | *Reference* | | *Reference* | |
|  | Generalized | 1.26 (0.66 to 2.41) | 0.48 | 1.20 (0.63 to 2.29) | 0.58 | 1.27 (0.44 to 3.63) | 0.66 | 1.29 (0.45 to 3.70) | 0.64 |
|  | None | 0.78 (0.46 to 1.31) | 0.34 | 0.81 (0.48 to 1.36) | 0.42 | 0.91 (0.39 to 2.08) | 0.81 | 0.96 (0.41 to 2.22) | 0.92 |
| Postoperative KPS | Focal | *Reference* | | *Reference* | | *Reference* | | *Reference* | |
|  | Generalized | 1.18 (0.58 to 2.40) | 0.65 | 1.08 (0.53 to 2.21) | 0.83 | 0.98 (0.32 to 3.03) | 0.98 | 1.01 (0.32 to 3.12) | 0.99 |
|  | None | 0.56 (0.32 to 0.99) | 0.047 | 0.58 (0.33 to 1.02) | 0.062 | 0.75 (0.30 to 1.79) | 0.51 | 0.80 (0.32 to 1.94) | 0.63 |
| Last FU KPS | Focal | *Reference* | | *Reference* | | *Reference* | | *Reference* | |
|  | Generalized | 1.58 (0.82 to 3.04) | 0.18 | 1.62 (0.84 to 3.13) | 0.15 | 0.73 (0.23 to 2.36) | 0.59 | 0.73 (0.23 to 2.36) | 0.59 |
|  | None | 0.98 (0.57 to 1.67) | 0.94 | 0.97 (0.57 to 1.66) | 0.91 | 0.89 (0.34 to 2.32) | 0.8 | 0.88 (0.34 to 2.30) | 0.79 |
| 1 HR = Hazard Ratio, OR = Odds Ratio, CI = Confidence Interval | | | | | | | | |  |
| ** All estimates were adjusted for patient age, tumor location depth and eloquence, adjuvant therapy ,frailty index, tumor volume, and pre-surgery KPS (was not included in "Pre-Surg KPS")* | | | | | | | | |  |

Supplementary table 4: Subgroup analysis by SE vs none-SE

| **Outcome** | **SE as Primary Manifestation** | | | | **SE as Follow-up Manifestation** | | | |
| --- | --- | --- | --- | --- | --- | --- | --- | --- |
|  | **Unadjusted Model** | | **Adjusted Model*** | | **Unadjusted Model** | | **Adjusted Model*** | |
|  | **HR** **(95% CI)***^1^* | **p-value** | **HR** **(95% CI)***^1^* | **p-value** | **HR** **(95% CI)***^1^* | **p-value** | **HR** **(95% CI)***^1^* | **p-value** |
| OS | 1.05 (0.46 to 2.43) | 0.9 | 1.09 (0.47 to 2.51) | 0.84 | 1.30 (0.79 to 2.12) | 0.3 | 1.30 (0.79 to 2.13) | 0.3 |
| Preoperative KPS | 0.47 (0.11 to 1.90) | 0.29 | 0.38 (0.09 to 1.59) | 0.19 | 1.72 (0.77 to 3.92) | 0.19 | 1.71 (0.77 to 3.91) | 0.19 |
| Postoperative KPS | 1.44 (0.31 to 7.42) | 0.64 | 1.05 (0.22 to 5.67) | 0.95 | 1.14 (0.49 to 2.67) | 0.76 | 1.12 (0.48 to 2.62) | 0.79 |
| Last FU KPS | 0.30 (0.08 to 1.12) | 0.074 | 0.32 (0.08 to 1.18) | 0.086 | 0.35 (0.14 to 0.92) | 0.033 | 0.36 (0.14 to 0.92) | 0.033 |
| 1 HR = Hazard Ratio, OR = Odds Ratio, CI = Confidence Interval | | | | | | | | |
| ** All estimates were adjusted for patient age, tumor location depth and eloquence, adjuvant therapy , frailty index, tumor volume, and pre-surgery KPS (was not included in "Pre-Surg KPS")* | | | | | | | | |
